# Changing the Underlying Conditions Relevant to Workplace Bullying through Organisational Redesign

**DOI:** 10.3390/ijerph20054373

**Published:** 2023-02-28

**Authors:** Yiqiong Li, Michelle R. Tuckey, Annabelle M. Neall, Alice Rose, Lauren Wilson

**Affiliations:** 1 UQ Business School, University of Queensland, St Lucia Campus, Brisbane 4072, Australia; 2 Centre for Workplace Excellence, UniSA Justice & Society, University of South Australia, Adelaide 5000, Australia; 3 School of Psychology, University of Queensland, St Lucia Campus, Brisbane 4072, Australia

**Keywords:** workplace bullying intervention, co-design, people management practices, qualitative and quantitative

## Abstract

In view of the discrepancy between anti-bullying strategies used in organisations and knowledge of bullying that is grounded in the international scholarly literature, the aim of this study is to implement and evaluate an intervention program specifically targeting the root causes of workplace bullying by identifying, assessing, and changing the contexts of people management in which bullying arises. The present research describes the development, procedures, and co-design principles underpinning a primary intervention that is focused on improving organisational risk conditions linked to workplace bullying. Our study evaluates the effectiveness of this intervention using deductive and abductive approaches and multi-source data. Specifically, our quantitative analysis examines changes in job demands and resources as a central mechanism underlying how the intervention takes effect and provides support for job demands as a mediator. Our qualitative analysis expands the inquiry by identifying additional mechanisms that form the foundations of effective change and those that drive change execution. The results of the intervention study highlight the opportunity to prevent workplace bullying through organisational-level interventions and reveal success factors, underlying mechanisms, and key principles.

## 1. Introduction

Workplace bullying, defined as negative or harmful behaviours that occur on a regular basis over a period of time resulting from the imbalance of power between the perpetrator and target [1], is a prevalent worldwide problem. Extensive evidence has documented bullying as a serious workplace health and safety (WHS) hazard that needs urgent attention. The Australian Government Productivity Commission estimated that the total cost of bullying to the economy is up to $36 billion every year in Australia, taking into account hidden and lost opportunity costs [REF]. According to Safe Work Australia [REF], the frequency of workplace bullying and harassment workers’ compensation injury claims per 100 million hours worked reached 17.5% in 2018–2019 (the most recent available data) compared to 6.6% in 2002–2003, whereas the rate for all mental stress claims declined over the same period (note that Safe Work Australia estimated that the 2018–2019 rate is likely to be higher than 17.5% once claim numbers are finalised). In addition, of all the mental stress claims, those arising from workplace bullying and harassment had the highest median direct cost (AUD $34,600), median time lost, number of claims, incidence rate, and frequency rate.

The deleterious outcomes of bullying are also documented in the research literature. Meta-analytical studies [2,3] have found significant relationships between workplace bullying and a range of health problems: anxiety, depression, post-traumatic stress, strain, psychosomatic symptoms, burnout, and physical health complaints. Costs to organisations include higher rates of absenteeism, greater intentions to leave the organisation, and lower job satisfaction and commitment [2]. Therefore, evidence-based strategies are needed to mitigate the costly impact of bullying and associated poor workplace mental health.

When seeking to address workplace bullying through evidence-based approaches, there is a discrepancy between the anti-bullying strategies used in organisations and knowledge of bullying grounded in the international scholarly literature. The academic evidence base highlights work and organisational characteristics as the principal contributing factors and recommends tackling these factors as a form of primary prevention [4]. In contrast, existing bullying prevention and response strategies, in practice, tend to focus on anti-bullying policies [5]; training to increase employees’ awareness, self-efficacy, and skills in response to negative behaviours [6]; or expert support in conflict resolution, mediation, and coaching [7]. These approaches are largely responsive rather than preventive, secondary and tertiary rather than primary, and focused on individuals rather than organisation-based. This bias is also evident in the published bullying intervention literature. For example, six of the twelve bullying intervention studies in Hodgins et al.’s review [8] did not tackle aspects of work and organisational design. Rather, they addressed individual-level factors only, consistent with the notion that bullying is routinely viewed as an individual-level interpersonal problem.

The ongoing emphasis on individual-focused strategies for mitigating bullying at work, despite robust evidence that organisational factors are the chief enablers, is a fundamental tension in the field. Several factors may play a role here; as researchers, we focus on two issues related to the knowledge base. First, general advice based on the existing evidence may not be specific enough to guide practical risk management efforts to prevent bullying and its associated harms. The most common bullying prevention recommendation from previous research is to improve work and organisational factors [9], for instance, by increasing role clarity and reducing ambiguity, ensuring that job demands are not excessive, and providing appropriate resources to perform the work. These sorts of generic recommendations are, however, difficult to translate into practice. For example, giving feedback is largely thought to be a critical way to clarify roles; however, meta-analytic findings demonstrate that the effectiveness of feedback-based interventions varies considerably [10]. It thus remains unclear how feedback should be given so that roles can be clarified. Similarly, in modern organisations, the pressure for high performance leads managers to demand more output from employees with the provision of fewer resources [11]. Against this backdrop, it seems rather trite to advise managers to ensure staff workloads are moderate and that there are adequate resource provisions.

Second, there is a lack of high-quality intervention studies to bridge the gap between existing research findings and tools that can be applied in practice. Although we know “what should work, in practice, we seem to lack sound evidence from intervention studies in real-life settings to support this” [12] (p. 255). Even though the knowledge base regarding bullying antecedents continues to expand [13], the evidence on prevention and response initiatives remains severely limited [8], especially regarding primary organisational interventions [14]. Moreover, rather than specifically addressing bullying, most intervention studies that are centred on improving social interactions in the workplace have focused on broad and general issues such as respect or dignity [15]. Perhaps the most well-known—civility, respect, and engagement in the workplace (CREW) [16,17,18]—addresses incivility (i.e., aggressive behaviours of low intensity) and may thus not be able to address the repeated, systematic, and threatening nature of exposure to bullying. Indeed, few targeted workplaces’ bullying intervention studies have been empirically evaluated [15,19,20].

In response to these two issues, the aim of this study was to implement an intervention program specifically targeting a reduction in workplace bullying by improving the underlying root cause organisational conditions, and evaluate it using mixed methods. Our study seeks to contribute to the workplace bullying literature in four ways. First, our intervention is a form of primary prevention. Rather than focusing on negative acts themselves (which are a product of problems in work and organisational systems), our study aims to tackle the underlying organisational conditions for workplace bullying by identifying, assessing, and changing the contexts of people management in which bullying arises [21]. These contexts offer clear focal points for risk management efforts to prevent bullying at the root of the problem, and solutions generated through co-design intervention offer clear avenues for bullying prevention. In this way, our approach shifts the focus of bullying prevention from individual employees to the organisational system and from reactive to preventative actions.

Second, our study investigates mechanisms through which the intervention influences workplace bullying based on the work environment hypothesis [22]. Studies framed by the work environment hypothesis have shown that bullying is associated with poor-quality work design, which is characterised by high demands and low resources [4]. However, it is also important to examine these underlying mechanisms in intervention research, which are manifested as mediating proximal effects [15,23]. Thus, our quantitative analysis examines changes in job demands and job resources as central mechanisms underlying how the intervention takes effect. Our qualitative analysis expands the inquiry by identifying additional mechanisms involved in the change process. Overall, building a more comprehensive understanding of these mechanisms can improve the design and implementation of interventions to ensure that they achieve their objective of preventing bullying, thereby helping to redress the relatively low levels of effectiveness seen so far within the literature [20].

Third, our quantitative analysis evaluates intervention effectiveness through changes in two types of outcome data: (1) self-reported exposure to bullying behaviours at the work unit level in a pre-test/post-test design; and (2) company internal data regarding complaints, employee advocacy, and customer satisfaction, through controlled comparison with stores operating under ‘business as usual.’ According to Escartín’s review [20], bullying intervention studies have tended to examine knowledge, attitudes, and perceptions as outcomes, and show limited and mixed findings regarding the effects of the intervention on bullying behaviour. Our evaluation responds broadly to the lack of evidence regarding the effectiveness of bullying interventions, especially primary organisational interventions, on exposure to bullying behaviour as a core outcome, and builds the evidence base beyond subjective criteria by analysing internal company data.

Fourth, we describe co-design as a core intervention process principle and outline how this principle is realised within different intervention phases. This aspect of our research represents a novel approach in the field and addresses the evidence gap regarding success factors for bullying interventions [19,20,24]. Such knowledge can guide researchers and practitioners about how to effectively design and implement a bullying prevention program beyond the content of the program itself.

## 2. Theoretical Framework for the Intervention

The dominant guiding framework for uncovering the antecedents of bullying has been the work environment hypothesis, which emphasises “characteristics of the psychosocial work environment as precursors of bullying” [22] (p. 476). Coping with a stressful psychosocial work environment erodes employees’ energy reserves and causes strain [25]. In turn, employees may act in ways that do not conform to workplace norms and expectations or perform their work less competently, which could be expected to place them in a more vulnerable position in terms of being bullied by supervisors and/or colleagues.

A central thread within this literature has been the study of psychosocial job characteristics as predictors, especially job demands (which require energy and effort) and job resources (which help achieve work goals, manage job demands, or help employees function to an optimal level). Early investigations showed that the targets of bullying report higher levels of job demands compared with non-targets [26,27], and likewise that levels of job demands are an important discriminating factor between departments with the highest, medium, and lowest levels of bullying [28]. Studies then examined associations between a range of different types of job demands (e.g., role ambiguity and role conflict, workload, job insecurity, and cognitive demands) and job resources (e.g., job control, social support) with bullying, as well as the interaction between job demands and resources on reports of bullying exposure. In general, findings have shown that the likelihood of bullying is elevated as job demands increase and job resources decrease [29,30,31,32], including for objectively measured demands and resources [33] and when predicting the use of bullying behaviours as a perpetrator [34].

The relationship between leadership styles and workplace bullying has been another focal point. The findings of these studies showed that some leadership styles, such as transformational leadership [35,36] and authentic leadership [37,38], are consistently associated with lower reports of bullying exposure. In contrast, destructive leadership styles tend to have a positive relationship with bullying exposure, particularly laissez-faire leadership [39], and non-contingent punishment and autocratic leadership [40]. More recently, some studies have failed to find significant leadership effects [41,42], and others have observed interactions between leadership and psychosocial job characteristics [43,44].

Our intervention research extends both of these lines of research. Our study is grounded in the work environment hypothesis by acknowledging the role of job demands and job resources in bullying, and the important influence of leadership; and takes a step further to look at when and how such job characteristics emerge within the context of the leadership process. Specifically, our intervention focuses on improving the contexts in which people management practices are carried out. Based on data from 342 real-life complaints, such contexts were identified as holding the greatest risk for bullying within organisational systems [21]. People management contexts refer to the micro-organisational conditions wherein supervisors organise and coordinate people and tasks to meet organisational goals within time and resource constraints [21].

The ongoing devolution of HR responsibilities to supervisors [45] highlights the importance of focusing on how these activities are performed. The effectiveness of how people management practices are carried out by line managers is considered the major cause of the gap between intended and perceived HR [46,47]. Line managers play a key role in translating formal HR policies into employee work experience through their day-to-day interactions with employees and their daily decision making to meet operational requirements. Even when intended HR practices are well designed, they require proper implementation to contribute to better firm performance and promote positive employee outcomes. In the worst case, the ways in which line managers carry out people management practices to elicit high performance from employees may be considered unreasonable or even abusive in the eyes of employees [11]. Despite its importance, the issue of how people management practices are actually carried out has largely been overlooked [48,49].

Our study builds on the overarching proposition that redesigning people management practices could reshape job demands (e.g., remove hindrance demands) or enhance job resources to lessen the negative impacts of job demands (e.g., provide social support to cope with workloads, change rostering allocations to better match capabilities to tasks) and/or foster growth and learning (e.g., provide positive feedback and recognition). In turn, redesigning people management practices could lead employees to appraise that job demands are reasonable and job resources are sufficient, and reduce their vulnerability to bullying. We thus anticipate that changes in job demands and job resources function as underlying mechanisms that link the redesign of people management practices to a reduction in bullying exposure.

## 3. The Intervention

Our intervention is designed to specifically address the risk contexts for workplace bullying, using co-design as a foundation. It builds on the theoretical framework of the work environment hypothesis and its scholarly evidence, with the proposition that healthy work design is the foundation for providing a safe and productive work environment. The intervention incorporates an evidence-based risk audit tool, which was developed by our team to diagnose the risk contexts for bullying through three studies with seven samples [21]. The diagnostic tool assesses the effectiveness of people management practices in ten organisational contexts, arranged in three domains: (1) coordinating and administrating working hours; (2) managing work performance, and (3) shaping relationships and the work environment. In the context of the intervention, the tool provides diagnostic data to guide action planning.

The intervention follows the problem-solving cycle recommended in the literature on organisational interventions, which typically consists of five phases: preparing for the intervention, screening to identify problem areas, action planning, the implementation of action plans, and intervention evaluation [50]. Organisational interventions are designed to improve the work environment *and* democratise the workplace; as such, incorporating structured participatory processes is considered fundamental to realising significant impact [51]. Co-design processes offer a powerful democratising opportunity [52], which we apply in each phase of the intervention as the underpinning process principle.

Co-design involves organised creative collaboration throughout a design process [53]. Originating in the participatory design movement of the 1970s, workers are recognised as experts in their own work experience, and researchers facilitate their involvement in the process of designing and implementing workplace changes by providing opportunities and tools for generating and expressing ideas [53]. The principles of co-design share similarities with those of participatory organisational interventions, in which improvement happens through “a collaborative approach where managers and employees jointly decide on the process (the design and implementation of the intervention) and the content of the intervention (changes to work policies, practices, and procedures)” [54] (p. 1). We integrate both of these literatures to inform our intervention.

By emphasising a collaborative approach between managers and employees, co-design enables both the active engagement and participation of employees and the support of managers at various levels, which, as several scholars argue, are critical elements of a successful intervention [50,55,56,57]. The co-design intervention process helps managers and employees to form a shared understanding of the problems and pain points [58] and supports them to co-create customised solutions to fit the context of their organisation and work unit [54].

Participation has four dimensions: content, process, directness, and goal [56]. In this intervention, employees participate in both the *content* (i.e., identifying problems in the work environment and making decisions about what needs to be changed) and the *process* of the intervention (i.e., discussing how changes can be made and leading their implementation). As for *direct* participation, all employees are invited to have input on the problems they experience in the risk areas via the risk audit tool. Although not all employees are invited to generate solutions at the workshop, which weakens the directness, several approaches are used to deepen involvement in each phase. Finally, by changing people management practices, our intervention removes some of the barriers within the organisation that could inhibit participation. Thus, the intervention does not only enable employee participation throughout each phase, it also creates an enabling work environment for promoting participation in the workplace more broadly, which is the ultimate *goal* of the participatory approach [56].

According to Nielsen and colleagues [51,54], participation has a range of benefits. It can (a) improve the substance of the intervention by utilising employees’ expertise and optimising the fit of the intervention to the work and organisational context; and (b) enhance intervention implementation by fostering buy-in, enabling dialogue between managers and workers, increasing exposure to the intervention, and smoothing the change process. Participation is also an intervention in and of itself. For instance, participatory processes can directly build resources at individual levels (e.g., autonomy, support, fairness, esteem) and team (e.g., sensemaking, cohesion) as well as for leaders (e.g., health-promoting leadership skills) and the organisation (e.g., capability to address psychosocial hazards).

The intervention additionally incorporates a range of success factors identified within the literature. These include: addressing work design at multiple levels [54]; integrating intervention strategies within existing organisational procedures and processes [50]; establishing senior management support through the commitment and provision of resourcing [55,59], obtaining middle manager support through commitment and involvement [55], and providing expert support to facilitate a structured and systematic intervention process [55,59].

## 4. Methodology

Our study applied mixed methods to evaluate the workplace bullying intervention. Quantitative data were used to investigate intervention effectiveness and examine how theoretical principles underpinning the intervention took effect in practice. In parallel, grounded theory was generated from qualitative data to identify additional mechanisms of change not comprehensively documented in the existing literature. The convergence of both quantitative and qualitative methods enabled us to capture the richness and complexity of the phenomena of interest [60]—changing the organisational conditions underlying workplace bullying. Commensurate validity [60] was upheld by assigning specialist team members to lead different aspects of the evaluation while working together throughout the research process.

### 4.1. Intervention Context

The intervention was implemented in 10 supermarket stores of a major Australian food retailer. Stores were nominated for the intervention by the organisation and were all located in one Australian state. Organisational data from the remaining 252 supermarket stores in the same state were used for the controlled comparison. Employees working in the 10 intervention stores held the roles of a team member, team leader, and manager in various departments (e.g., front-end, fresh produce, grocery, deli, bakery, night fill, and office). Two local HR partners supported the stores throughout the intervention process (e.g., booking workshop venues, attending the workshops, and checking in with stores during implementation). The intervention process was supported more generally by two members of the central HR team (e.g., sending out diagnostic and evaluation surveys, obtaining organisational data for analysis, and arranging charge-off codes to cover store members’ time invested in the survey and workshops).

### 4.2. Intervention Phases and Data Sources

The intervention was implemented in five phases: preparation, diagnosis, solutions, implementation, and evaluation. Each phase is outlined in more detail below, and the data sources used for the quantitative and qualitative aspects of the evaluation are mapped in Table 1 in connection to the phases.

*Preparation*. The research team worked closely with central and local HR staff, meeting weekly throughout the intervention. This group collaborated to coordinate stakeholders; brief senior national leaders of the organisation; brief above-store managers in the operations chain; liaise with store managers to promote intervention participation; communicate the purpose, progress, and anticipated outcomes of the intervention; and safeguard the ethical aspects of research. The nature and timing of the intervention were tailored through these meetings.

*Diagnosis.* All team members, team leaders, and managers over the 10 stores were invited to complete the diagnostic survey during work hours online via the store computers. The survey was open for two weeks, with voluntary participation. Responses were confidential and linked to those at the evaluation phase by a self-generated identification code. Following the survey, a diagnostic risk profile graph was generated for each store by averaging the scores in each risk area measured on the risk audit tool. Across all 10 stores, the three lowest-scoring contexts of people management identified via the tool were: recognising and rewarding job performance, managing tasks and workload, and managing underperformance. Providing training and professional development, promoting mental health and well-being, and interpersonal and team relationships were identified as additional risk areas in some stores. The highest-rated area of people management practice across all stores was maintaining a safe working environment.

*Solutions.* Four co-design solution workshops of a 3.5 h duration were conducted over three days in October 2020. Each workshop included team members and store managers from two or three stores, which were grouped according to geographical location. Up to 15 participants from each store, across a range of employment levels and departments, were invited to participate. At the outset, the facilitators from the research team framed the workshop as a problem-solving and future-oriented initiative to identify changes that could make their store a better place to work. The facilitators positioned the participants as experts in their own store work environment and explained their key role in providing expert insights on what would work well and how to make things better to support their work in-store. In order to neutralise the inherent store operational power hierarchy, the facilitators positioned the role of store managers as listening and learning from the other store team members.

Workshop participants collaborated on two co-design activities in groups of approximately five; both activities focused on the three or four risk contexts that were rated as least effective according to the diagnostic profile for their store. First, participants identified the pain points in each risk context and the underpinning policies, procedures, and practices. Using this information to guide them, participants then developed interim and longer-term solutions to address the challenges in the risk contexts, thereby improving the way each people management practice was conducted.

*Implementation.* The solutions devised in the workshops were implemented in the stores between late October 2020 and late June 2021 (i.e., approximately eight months). Store managers initially formed an action plan for the store, with input from other store team members, to prioritise and plan how to implement their solutions. Crucially, implementation was co-led by change champions—key team members, team leaders, and department managers (who had typically attended the workshop)—rather than solely relying on store leaders [61]. One-hour monthly check-in meetings (i.e., a series of collaborative review sessions) were facilitated by the research team to enable representatives from each store to learn from each other by sharing progress and knowledge (Steen, 2013), solve implementation challenges, and work out ways to keep the project alive.

*Evaluation.* In the final phase, all store team members and managers were re-invited to complete the diagnostic survey. The survey was again open for two weeks. Internal company data were also obtained for: (a) complaints regarding employment relations matters, workplace grievances, and workplace bullying; (b) employee advocacy indicative of culture and employee engagement; and (c) customer satisfaction. Following the conclusion of the intervention, two focus groups and two interviews were conducted via an online platform with participants from across the 10 stores and from the supporting HR team.

### 4.3. Quantitative Methodology

#### 4.3.1. Participants

A total of 399 and 339 respondents across the 10 stores responded to the Time 1 (August 2020) and Time 2 (July 2021) surveys, respectively. Approximately 90% of respondents were under the age of 50 years, and the average organisational tenure of employees was 7.67 years (SD = 8.04). Participants were typically employed on a permanent full-time (Time 1 = 35%, Time 2 = 32%) or part-time (Time 1 = 34%, Time 2 = 46%) basis. About 50% of participants at Time 1 were female, 42.1% were male, 1.5% non-binary, and 6.8% preferring not to say, with a similar pattern at Time 2 (50.7% = female, 40.6% = male, 0.6% = non-binary and 8.1% = prefer not to say).

#### 4.3.2. Quantitative Survey Measures

*People management practices*. A behaviourally anchored rating scale developed by our team [21] was used to assess employees’ perceptions of people management practices in ten risk contexts. Participants were asked to provide an overall effectiveness rating by using a sliding scale (equivalent to a 10-point scale) to indicate how effectively people management practices were typically carried out in their work area. Positioned beside the scale were behavioural indicators, where participants selected the indicators most relevant to their workplace in order to guide their overall ratings. Finally, a comments section was provided where participants could optionally elaborate on their ratings by providing written information to contextualise their responses.

*Exposure to Workplace Bullying*. Notelaers and colleagues’ Short-Negative Acts Questionnaire (S-NAQ) [62] was employed to assess employees’ perceptions of exposure to workplace bullying through measures of person-oriented, work-related, and social exclusionary bullying behaviours. Responses were rated on a 5-point frequency scale (1 = Never, 2 = Now and then, 3 = Monthly, 4 = Weekly, 5 = Daily). An example from the 9-item scale was ‘Someone withholding information which affects your performance’. This measure demonstrated good internal consistency (α = 0.90 at Time 1).

*Job Demands* were measured using the Demand Induced Strain Compensation Questionnaire English Version 2.1 (DISQ 2.1) [63] to assess cognitive (e.g., ‘I have to make complex decisions at work’), emotional (e.g., ‘I have to do a lot of emotionally draining work’), and physical demands (e.g., ‘I have to perform a lot of physically strenuous tasks to carry out my work’). Responses were recorded on a 5-point Likert-type scale (1 = Never or very rarely, 5 = Very often or always). The coefficient α for this measure at Time 1 was 0.91.

*Job Resources*. The DISQ 2.1 was also used to assess cognitive (e.g., ‘I have the opportunity to take a mental break when tasks require a lot of concentration’), emotional (e.g., ‘I feel esteemed at work by others’), and physical work resources (e.g., ‘I am able to decide what posture I use to perform physically strenuous tasks), rated on the same 5-point Likert-type scale. Coefficient α at Time 1 was 0.91.

#### 4.3.3. Internal Company Data

*Internal complaints.* The number of internal complaints lodged by employees regarding employment relations matters, workplace grievances, and workplace bullying was provided by the organisation for the intervention and non-intervention stores as a whole. The average number of complaint cases for each of the two cohorts (intervention versus non-intervention) was calculated by dividing the total number cases for the cohort by the number of stores in the cohort.

*Employee advocacy*. Employees were asked to rate the extent to which employees would recommend their organisation and their store as a good place to work on a 10-point scale from 0 to 10. Advocacy scores were measured as a net promoter score (i.e., the percentage of employees who rated 9 or 10 subtracted by the percentage of employees who rated below 7).

*Customer satisfaction.* Customers who recently shopped at a store were invited to rate the extent to which they would recommend the store as a good place to shop on a 10-point scale from 0 to 10. Similar to employee advocacy scores, customer satisfaction scores were also measured as a net promoter score using the same calculation method.

#### 4.3.4. Quantitative Analysis

The quantitative analysis utilised the data sources outlined in Table 1. The intervention in our study is a group-level intervention, focusing on changing people management practices at store and department levels so that analysis could be conducted at both of these levels. (Results of an individual-level analysis using data from the 74 employees who responded at both time-points is available from the authors on request). Survey data from all respondents were aggregated to the store level to examine the effectiveness of the intervention using paired sample t-tests. Time 1 respondents worked in 58 departments (across the 10 stores), and Time 2 respondents worked in 56 departments (across the 10 stores). Those employees who responded at both time points were spread across 54 departments, 34 of which had more than 2 participants at both time points. These 34 departments, which had 261 and 246 participants at Time 1 and 2, respectively, were used for department-level analysis. (The remaining 19 departments were removed from the department-level analysis). At the department level, we examined the effectiveness of intervention using paired sample t-tests and investigated the mediating effect of job demands and resources in the relationships between people management practices and the reduction in bullying exposure by estimating the indirect effects of mediation analysis using the bootstrap procedure.

### 4.4. Qualitative Methodology

#### 4.4.1. Qualitative Design

We engaged in abductive, theory-refining research adopting Gioia and colleagues’ framework [64] for grounded theory methods. Grounded theory is well suited to the aims of the qualitative component of our study due to its central features: asking theoretically orientated questions, following an iterative process, and generating results through data-theory interplay [65]. Compatible with grounded theory methodology, a qualitative methods design was adopted to allow for the exploration, extension, and refinement of understandings of mechanisms for change, including but not limited to the theoretical framework which informed the intervention design [66].

#### 4.4.2. Qualitative Sampling

The qualitative evaluation used the data sources outlined in Table 1. Our sampling strategy adopted a modified approach to Gioia and Colleagues’ [64] grounded theory sampling methods for theory construction and development over three waves of qualitative data collection. The first wave used purposive sampling, inviting 40 employees at various levels from across the 10 stores to participate in focus groups in order to capture a broad range of experiences. As part of the iterative approach, which is considered fundamental to grounded theory methods [64], theoretical sampling was then used again to shape the second wave of data collection, which sought to interview key HR personnel who had supported the intervention process locally both at the store level and centrally. As new properties that could further elaborate on theory were still emerging from the data analysis during the second wave, a theoretical sampling frame was devised for a third wave. In the third wave, the records from check-in meetings that were held during the implementation phase were accessed and iteratively analysed. Although these records were taken earlier to support successful implementation, they provided rich data during the qualitative evaluation to understand the change process in situ.

#### 4.4.3. Qualitative Materials

In Wave 1, semi-structured focus group and interview schedules were developed by the research team, drawing upon the results of the Time 1 and Time 2 survey analysis and the process evaluation literature [23,67]. Two focus groups were held with a total of 10 participants (team members = 3, department managers = 4, assistant store managers = 2, and store managers = 1) from five of the ten stores. The focus groups were facilitated using a secure online platform and ranged in duration from 46 to 48 min. To gain further insights into the mechanisms of change at a more holistic level, in Wave 2, two semi-structured interviews were also conducted with HR employees who were involved with the intervention. Interviews and focus groups were recorded using the online platform and transcribed by a professional transcription service in preparation for analysis. Wave 3 analysed data from the six implementation meetings, which were held at roughly monthly intervals during the implementation phase. Each meeting involved store leader and store team member participants from across the 10 stores.

#### 4.4.4. Qualitative Analysis

Throughout the qualitative analysis, the fourth author adopted an interpretive approach, allowing for the results to be grounded in the data in a way that reflects participant experiences *and* permits abstraction to theoretical models without reference to specific pre-existing theories [68]. Coding and the analysis of the qualitative data were undertaken in three non-linear phases of constant comparison, in accordance with Gioia and Colleagues [64]. Simultaneous with data collection, the fourth author analysed waves of qualitative data by adopting a constant-comparative analysis coding strategy, continually refining and recalibrating first-order concepts, and developing second-order themes. To support analysis, NVivo qualitative software (version released March 2020) was used to organise the codes and record memos and notes on theoretical and analytic aspects of the data. Once the data from Waves 1 and 2 were coded into initial concepts and suggested second-order themes, the fourth author met with the second author to review and discuss one-quarter of the initial concepts. An analysis of the Wave 3 data was undertaken using the same method of constant comparative analysis, and the initial concepts and second-order themes were revised to capture all three data waves.

Four members of the research team then met in person on four occasions to advance the data analysis and develop a theoretical model of the results. As the data were further abstracted into the final aggregate dimensions, members of the research team with superior theoretical knowledge played a larger role in the analysis. In the first meeting, the team reviewed the initial concepts and their associated raw data, revising the concepts that best reflected the research team’s interpretations of meanings in the data. The next meeting focused on the development of second-order themes, which the research team openly reviewed to ensure that a range of perspectives and interpretations of the data were considered [64]. Drawing upon memos and notes from the meeting, the concepts and themes were then revised further by the fourth author. Following this, the research team met again to reach an agreement on second-order themes and aggregate dimensions. Once the aggregate dimensions had been defined, a data structure providing a visualisation of the progression from raw data through to aggregate dimensions (see Table S1 in the Supplementary Materials for the qualitative data structure) was shared across the research team for review and comment. The research team met again twice, firstly to review and refine the aggregate dimensions for their boundaries and clarity of meaning, and then to develop theoretical models which would illustrate the findings of qualitative methods and to discuss the ways in which the findings extended, contrasted, and aligned with pre-existing theories in the literature.

## 5. Results

### 5.1. Quantitative Findings

#### 5.1.1. Aggregation of Data to Store- and Department-Level

Before aggregating individual responses to the store or department level, interrater reliability r_wg(*j*)_ and intraclass correlation coefficients [ICC(1)] were calculated for each measure to evaluate the aggregation (see Table 2 for results). The r_wg(*j*)_ agreement index represents a within-group agreement, and ICC(1) represents the amount of variance in individual responses, which could be explained by group membership. Using LeBreton and Senter’s [69] cut-off and effect guidelines, the r_wg(*j*)_ results exceeded the cut-offs (>0.70) for all measures at both the store level and department levels at the two time points. Furthermore, the measures of people’s management practices and job demands met the ICC(1) cut-offs of 0.07 at both the store level and department level at two time points [69]. This suggests that individuals shared similar perceptions regarding how people management is performed and regarding the extent to which their jobs are demanding within their store and department. These results provide sufficient evidence to justify the aggregation of people management practices and job demands at the store or department level.

Conversely, the ICC1 results for the bullying measures at Time 1 and Time 2 failed to meet the ICC1 cut-off at the store level or department level. This result indicates that individuals experienced differing perceptions of bullying exposure within and between stores and departments. We also found that the measure of job resources failed to meet the ICC(1) cut-off of 0.07 at the department level at Time 1 even though it exceeded the cut-off off 0.07 at Time 2; we could not determine a meaningful reason for this pattern. We nonetheless aggregated job resources at the department level for the mediation analysis because the r_wg(*j*)_ results were acceptable, and the measure of job resources at Time 2 was the key measure in the analysis (whereas the Time 1 measure was used only as a control variable).

#### 5.1.2. Descriptive Statistics

Table 3 presents the means, standard deviations, and intercorrelations of the quantitative measures. At the store level, with such a small sample size, measures tended to be significantly correlated only with variables measured at the same time point. Of note, people management practices were significantly and negatively related to workplace bullying behaviour at Time 1 (*r* = –0.69, *p* < 0.05) and Time 2 (*r* = –0.87, *p* < 0.01). At the department level, Time 1 workplace bullying behaviour was negatively correlated with people management practices (*r* = –0.58, *p* < 0.01) and job resources (*r* = –0.60, *p* < 0.01), while also positively correlated with job demands (*r* = 0.65, *p* < 0.01). At Time 2, workplace bullying was negatively correlated with people management practices (*r* = –0.43, *p* < 0.05) and positively correlated with job demands (*r* = 0.58, *p* < 0.01) while not significantly related to job resources (*r* = –0.32, *p* = n.s.). Time 2 job resources were positively correlated with people management practices (*r* = 0.46, *p* < 0.01).

#### 5.1.3. Intervention Effectiveness

Paired-sample t-tests were employed to investigate the effectiveness of the intervention in reducing workplace bullying exposure. The t-test results revealed that exposure to workplace bullying behaviour decreased significantly over the course of the intervention at the store level (*M* = 0.25, *SD* = 0.20), 95% CI [.40, 2.10], *t*(9) = 4.01, *p* = 0.003; and at the department level (*M* = 0.24, *SD* = 0.44), 95% CI [.19, 0.91], *t*(33) = 3.21, *p* = 0.003.

**Figure 1:**
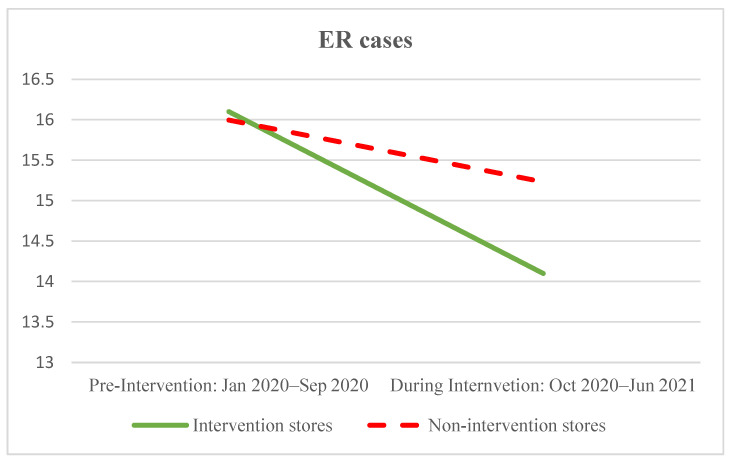

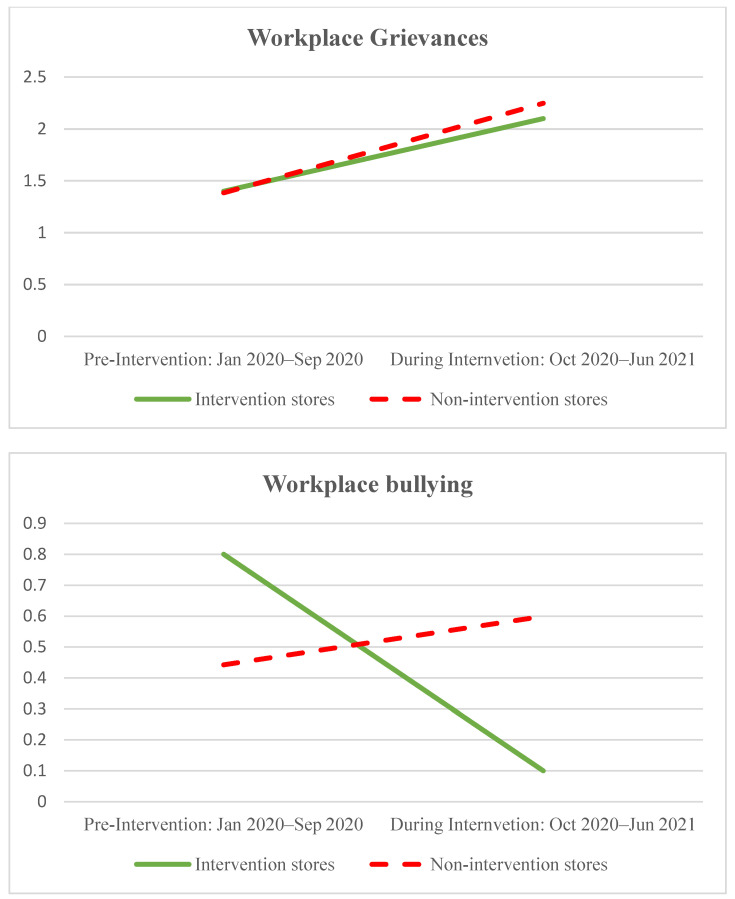
A comparison of company internal complaint records between intervention stores and stores of the same organisation that were not exposed to intervention.

The internal company data regarding complaints, employee advocacy, and customer satisfaction also illustrate the effectiveness of the intervention at the store level relative to comparison stores, as shown in Figure 1. Workplace bullying complaints lodged by employees in the intervention stores decreased from an mean of 0.80 cases per store during the pre-intervention period (January–September 2020) to 0.10 cases per store during the intervention period (October 2020–June 2021), whereas the 252 comparison stores recorded an increase from 0.44 to 0.60 cases on average during the same period of time. Employment relations cases decreased by a mean of 2.00 cases per store in the intervention stores, in comparison with a reduction in 0.76 cases per non-intervention store, on average. The number of grievances increased for both groups, though the mean increase was lower for the intervention stores (from 1.40 to 2.10) compared with the comparison stores (an increase 1.38 to 2.25 cases per store).

**Figure 2:**
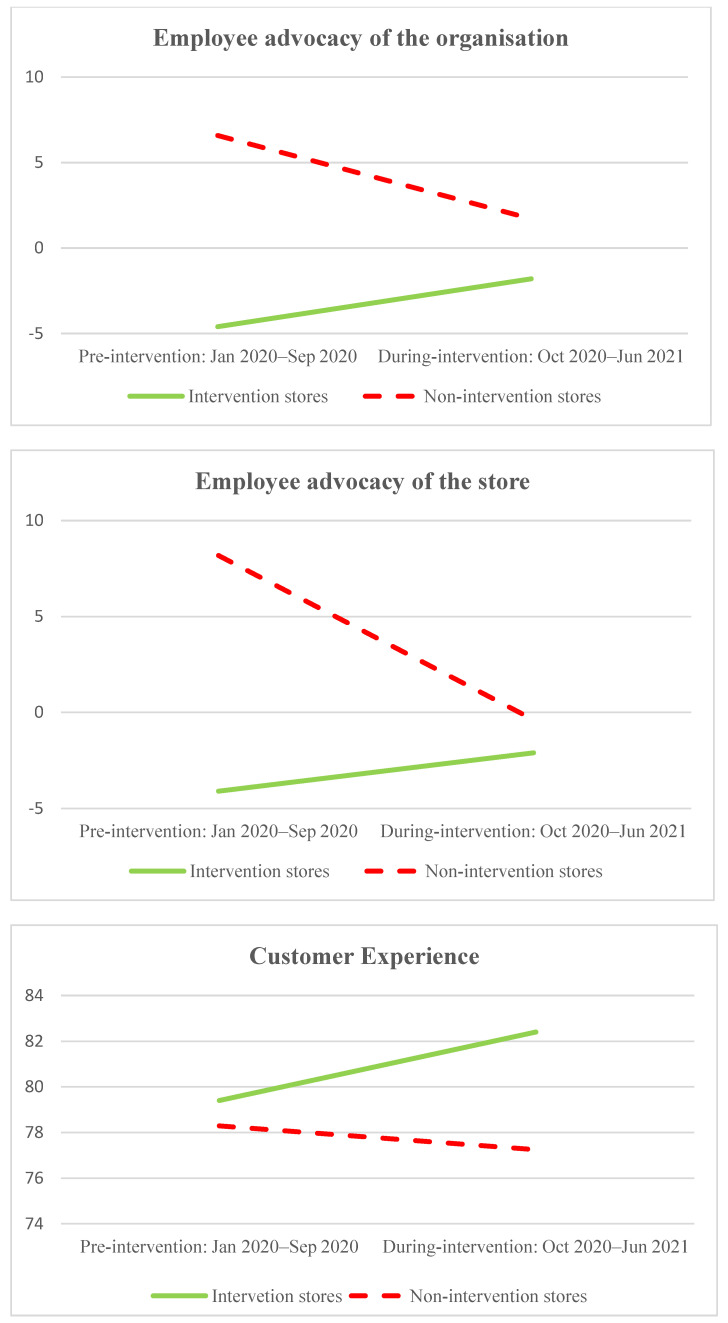
A comparison of employee advocacy and customer satisfaction scores between intervention stores and stores of the same organisation that were not exposed to intervention.

Similarly, intervention stores reported an increase in employee advocacy and customer experience scores over the intervention period, while non-intervention stores reported a decrease (see Figure 2). Specifically, in the intervention stores employees reported an increase in advocacy for their organisation and their store (from −4.60 to −1.80 and from −4.10 to −2.10, respectively), while the non-intervention stores experienced a reduction in both types of advocacy (from 6.58 to 1.71 and from 9.18 to −0.39, respectively). Customers who had shopped recently in the intervention stores reported an increase in customer experience (from 79.40 to 82.40), while the non-intervention stores experienced a reduction (from 78.29 to 77.25).

#### 5.1.4. Intervention Mechanisms

We ran mediation analyses to explore the role of job demands and job resources in transmitting the effects of the intervention, using data aggregated to the department level. At this level, Time 2 people management practices were positively related to Time 2 job resources (*b* = 0.21, *p* = 0.005) and negatively related to Time 2 job demands (*b* = −0.23, *p* = 0.004), while controlling for Time 1 resources and demands, respectively (see Table 4). Time 2 workplace bullying was positively related to Time 2 job demands (*b* = 0.44, *p* = 0.000) while controlling for Time 1 workplace bullying (*b* = 0.14, *p* = 0.351). The indirect effect between people management practices and workplace bullying via job demands was significant (indirect effect = −0.10, Boot SE = 0.04, 95% CI [−0.019, −0.02]) and consistent with the premise that job demands play a mediating role. However, workplace bullying was not related to job resources at Time 2 (*b* = −0.21, *p* = 0.114), thereby providing no support for the mediating role of job resources in realising the changes in bullying exposure.

We also performed a supplementary mediation analysis which removed job demands and only included job resources as a mediator. In this analysis, workplace bullying was significantly related to job resources (*b* = −0.24, *p* = 0.045), however, the indirect effect between people management practice and workplace bullying via job resources was still not statistically significant (indirect effect = −0.05, Boot SE = 0.03, 95% CI [−0.11, 0.01]).

### 5.2. Qualitative Analysis

**Figure 3:**
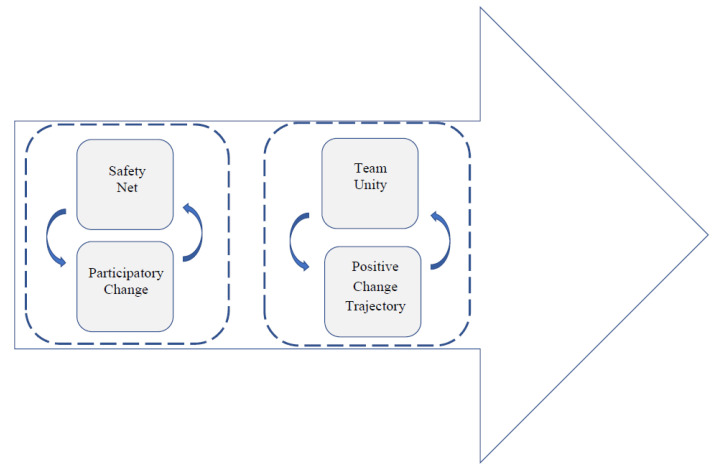
Process model of the dynamic mechanisms of change.

The analysis of the data using grounded theory methods generated four aggregate dimensions—safety net, participatory change, team unity, and positive change trajectory—which together represent a dynamic set of mechanisms through which our intervention reduces the risk of workplace bullying (as illustrated in Figure 3). Each of these change mechanisms involves a multi-layered process that includes the team, department, leader, and store dynamics, as well as mental models and enactment to promote change. Safety net and participatory change form the foundations of the change process, onto which team unity and positive change trajectory can be grounded in order to support the execution of change. Notably, while communication was not a stand-alone dimension or a separate mechanism, it was a feature of the data. Aspects of communication were identified as acting in service of each of the four aggregate dimensions in different ways. For example, communicating commitment and adopting a whole-person approach to communication are key components of the safety net. Participatory change was facilitated by top-to-bottom communication along the formal operational hierarchy. Opening up lateral communication channels promotes team unity, and communication enables involvement and recognition, which promotes a positive change trajectory.

#### 5.2.1. Safety Net

Foundational as a change mechanism, safety net involves the establishment and ongoing provision of a work environment that feels safe for team members and store leaders to invest in the change process, be open to connecting with others and sharing ideas, and collaborate with a broader range of co-workers across different departments and levels. The safety net is unique as a dimension in that it is largely the role of store leaders to create the trust needed for team members and team leaders to open up, share, and invest. Given the power structures inherent within store operations, team members are typically at the end of work design rather than playing a role in shaping it, but this intervention process offers them an opportunity to be involved more proactively in influencing what happens in their store.

*“However, just being involved in the workshops and being involved in a survey where people are actually listening and responding, where group managers and store [managers], and [HR] partners are paying extra attention, where we’ve got an extra project team and me and other people going in and checking in on them and asking questions and providing support. I think yeah, I think that’s the most valuable part is just they see we’re trying and we see, they see we care. And then on the back of that they have that attitude towards their own team, so a lot of the time the store managers see we’re trying and they’re able to go back to their team and have the same kind of attitude and just show they’re trying.”*

HR Partner

To experience the safety net, store team members needed to have clear signals from leaders above and within the store—those who usually set the store agenda in a top-down process—that it was safe to care about and invest in making changes because meaningful action would be taken based on their input. The signal that these leaders were committed to ensuring resource change was set up as part of the foundation for the safety net and the change process more broadly. When team members perceived that their organisation cared about them, as evidenced by the words and deeds of their leaders, they interpreted that as a signal that it was safe to care about the change plan and become willing to open up and take risks because they anticipated a meaningful outcome.

*“and there was a lot of seniority in the room as well and we had [HR partner] and a few other higher ups and what not, so we had that kind of, I guess that safety net where we’re seeing our higher ups there, obviously it’s interesting, obviously it’s something new coming.”*

Team member

*“I’m in total agreement, yeah I think that’s exactly right. I think I myself, definitely if I see a higher up there, I definitely take something more seriously, because it’s kind of hard to tell what is going to be the next thing we need to focus on, because so many different initiatives and so many different things happen, all year around this business is changing an incredible amount, really, really quickly, at pace, and apparently that’s what they call it, but it’s—yeah, seeing higher ups there [allows me to invest]”*

Manager

While the communication of commitment is an important component for establishing the safety net, the dimensions of the safety net extend beyond espousal to demonstrable activities. Otherwise, the safety net goes missing amongst the daily workload and time pressure, and leaders remain focused on the negatives. Team members are alert to managers who take the opportunity to enact their values and prioritise the intervention process, acting on commitments to improving employee well-being in an embodied way. When store leaders walk the talk and are willing to go to new places, then team members become willing and brave enough to journey where they have not been before, feel safe to feel hopeful, and invest greater energy into the change process.

*“so, the store managers understand that part of the problem is that people don’t see action and they don’t feel like they’re being heard. So just a shift to go to actually try things and show the team that they’re listening, and that’s what’s kind of shifted them to just trying little things and implementing them rather than waiting and sitting back. And you know, they [managers] might be planning in their office what to do about it, but if there’s no action the team member doesn’t know and they just feel unheard. I think that’s, to me that’s the difference, they’re trying to show to the team that they’re being listened to.”*

HR Partner

Adopting a person-centred approach is also part of creating safety in order to make changes in a participatory way. In particular, a person-centred approach plays a key role because it takes into account employees as individuals, giving thought to their well-being when making changes, and that helps the team members align with organisational goals. Team members experience the sense that they can bring their whole selves to work when they feel valued, and trust store leaders to take their needs into account. This safety net provides space to discuss the uncomfortable things that need addressing

*“…we’ve just kind of been able to let team open themselves up a little bit more to us and we’ve been kind of able to ask what’s up, what’s wrong, and people have been telling us, as opposed to kind of that cold shoulder and the feeling of nervousness that team members were feeling.”*

Manager

*“We have really been pushing team leaders and managers to manage people, not manage the job. Focus on speaking to people and working with people on the way that works best for them. Giving coaching on how to address challenges and complexities.”*

Manager

Adopting a whole-person approach—a way of working around and *with* the employee and not *on* or *over* the employee or even *for* the employee—promotes an alignment between and within team members, who then feel able to bring their whole selves to work. Awareness of and engaging with a safety net supports team members’ whole selves in becoming involved in the organisational change.

#### 5.2.2. Participatory Change

Along with a safety net, participatory change is identified as a foundational mechanism for change. This dimension encapsulates several aspects of participation that inherently activate the change process, especially by facilitating alignment between store members: listening to the team, empowering team members, and manager involvement for change. Part of achieving change requires store members to engage in and drive the process in an active way, through which they come to own the change process. Wholistic in nature, managers, teams, and individuals all play an essential role in participatory change—each party becomes involved and works collaboratively in making changes towards the ultimate goal.

*“my selling point [for investing in change] that I spoke to everyone, and why a lot of the people that are around me were hyped about it, was that I just said this is for us, this is for our future, this is for us working at [the organisation] in 10–15 years, like not all of them, obviously but those of us who are still going to be with the company in leadership positions, in whatever, that the company is going to start listening and showing people that this is a positive change and we’re moving forward, so they’ve got to be part of it, it’s so important to be a part of these changes…”*

Manager

Managers hold formal organisational power within the store, and when they are actively involved in the change, this sends a safety signal (as seen in the safety net). However, over and above the signalling value, managers are decision makers, resource controllers, and resource generators in organisations. Managers have responsibilities and accountabilities, which means they have to be involved in fostering effective change but, equally, they have to distribute some power to team members for a successful change process

*“… and also empowering the team by making just a team member the champion I think would just change the culture on the shop floor level, like on the team member level because they see that one person being able to make changes and they’ll know that they can do it too.”*

Manager

As organisations are hierarchical in nature, team members need to be empowered to foster holistic participation. Team participation has two elements: team members first need to have a voice and feel heard, and then they need to act. Team members want their managers to lead the way and take action toward positive change. In parallel, there is an empowerment process where team members are supported to bring up ideas, take responsibility, and can lead and develop some agency. Thus, while organisational hierarchies remain, they become less rigid through listening to team members, sharing communication from the top, and empowering bottom-up action. Rather than a top-down or bottom-up change process, our analysis of the data highlights the co-occurrence of top-down and bottom-up processes as a key mechanism of change.

*“On the back of recognition initiatives, the team gave feedback asking the store manager to recognise their [leaders] more, so that ripples down to the team, so role modelling that recognition is starting that this week”*

Manager

*“We’re seeing that, right? Like they’re, it’s not ‘oh we have this issue in store, we’re so annoyed’, it’s ‘we have this issue, let’s ask the team how we can fix it’—like a solution to be solved rather than a problem.”*

HR Partner

The concurrence of top-down and bottom-up change processes occurring through effective leadership and power distribution promotes increasing alignment in understanding the problems, the potential solutions, and how everyone can act together. In turn, this shared picture minimises competing goals and competition for resources and instead fosters reinforcing efforts. The participatory piece facilitates a wider range of parties that are involved in working more collaboratively in making changes towards the end goal. The interplay and receptivity between the bottom-up and top-down processes reinforce participatory change.

#### 5.2.3. Team Unity

Team unity, encompassing a dynamic set of related unifying processes, was identified as a key mechanism of change execution. Enacting team unity includes and extends beyond the communication of unity as a value. This dimension also incorporates opportunities to practice team cohesion through engagement in activities that reduce the barriers of the formal store hierarchy and the silos between departments. For example, cross-training was a common strategy used to enhance how team members from different departments work with each and towards the same store-level goal. Extending beyond an espoused attitude, team unity represents both the mental model of “one team” with the practice of working as one team.

*“Yeah, one whole team not just your—like you’re a team player not a department player, you’re about the whole store, not just your department; that’s a big difference I’ve noticed. Yeah, that’s us.”*

Team member

*“I think we were kind of not really a team, but we were like several teams, but now I think we’ve had that opportunity to really grow and develop, and send people across and cross-train people, so it’s now more of that one team mentality as well.”*

Manager

Unique to team unity, among the four aggregate dimensions, is its focus on changing interpersonal and inter-team relationships. Supported by a sense of safety in bringing their whole selves to work and being encouraged to participate in change, store members orient themselves toward shared goals in an open way in which genuine relationships are fostered. Store members support each other more widely and recognise these efforts across the store. They become involved in helping each other out more willingly, creating the sense that they have each other’s backs.

*“Yeah, I feel the vibe in the whole store is a lot supportive and everyone recognises each other’s hard work no matter what level you’re at; so doesn’t matter if you’re just a team member, noticing another team member working really hard and you’re like great job there [name], or whoever; everyone’s just a lot more supportive and we just have each other’s back a lot more […] we’re more like a family.”*

Manager

*Today was a good example: we made some changes in [a department]. To see the whole team help without being asked was so humbling, to see it happen automatically.*

Manager

These factors, in turn, facilitate an alignment of thinking and understanding, as well as a practical approach to achieving goals. Through this process, team members build stronger mental models of “we” when considering themselves in relation to their work (moving away from “I”). In this way, team unity is effective in reducing vertical and horizontal barriers to task-related interactions and relationships.

*“There shouldn’t be any team members in store now that you don’t feel comfortable to say hello to. Massive improvement there. Especially with team members that went to the initial session [workshop]—it brought them out of their shell.”*

Manager

Communication, a factor that operates in service of each of the aggregate dimensions, is instrumental in supporting team unity. The communication of clear plans for taking action promotes a unified team vision.

*“Our new catch phrase is: ‘More hands make light work.’ So we have started swarm filling, […] and there’s new emphasis on working as ‘one team,’ and we’ve started doing department huddles and took department out of store to hash out their issues. They weren’t being communicated to about the WHYs and they needed that!”*

Manager

The sharing of expertise, as well as the delegation of tasks and cross-training, are ways in which communication promotes the improvement of functional flexibility. Using communication in these ways, in concert with opportunities to experience and practice team cohesion, promotes the breaking down of barriers, creates experiences of team alignment, and changes interpersonal and inter-team relationships.

#### 5.2.4. Positive Change Trajectory

The positive change trajectory dimension is a dynamic change execution mechanism. Supported in part by the safety net and opportunities for participatory change, the trajectory begins with a liberating framing that promotes a sense of possibility for change among team members. From this point of possibility, a positive change trajectory builds with a change-orientated mindset and positive affect that can be fostered with small acts that propel change forward in a cumulative process.

*“Like the other stores [are seeing], the little things make the difference—thank yous, an open-door policy.”*

Team member

Awareness of the change in store culture is one example of a practical aspect of the trajectory: stores start with small and quick wins, and team members then notice the impact of small changes (i.e., culture starts to change). Eventually, more (critical and major) changes are built up based on the small changes and involve a wider range of team members. In this way, team members who may not feel comfortable speaking up at the launch of a change intervention experience or the feeling of the culture changing within their store become willing to make a change and even initiate a change.

*“…there was a lot of team members who had been with the business for quite some time, so it was kind of nice to show them the progression of the business, especially regarding mental health and being comfortable with your team and being comfortable with your leaders, especially.”*

Manager

Management communication regarding change is an ignition switch to the dynamic nature of this dimension. Our data suggest that awareness is important for overcoming team members’ apprehension, based on limited change outcomes in previous initiatives across the course of their working lives, and converting it to optimism through the positive change trajectory. Therefore, a key learning for managers is that a foundational aspect of a positive trajectory comes from creating awareness of change efforts and results among team members.

*“What I’m saying is that like with this push and seeing such a positive result and a positive, and noticing the whole team light up when you talk about mental health and how you can better assist them. It’s in our—I guess it’s in my best interest as a leader, and the project’s best interest to actually put in that extra bit of effort, because it’s the only way you’re going to get the ball rolling.”*

Manager

The importance of consistent effort together with the ongoing visibility of the effects are vital intervention ingredients, as one manager explained: “*stick with it, these things don’t happen overnight, keep chipping away at it*.” Within a positive trajectory, not everything has to go smoothly; the ongoing change process becomes most important.

*“And even if it doesn’t work and we’ve heard examples of where they’ve implemented stuff and it didn’t work out, at least they can, they showed to the team that ‘hey, we tried it, we listened to you, didn’t work, if you have other suggestions let us know and we can try again’.”*

Operations

At a local level, team members begin to do little things, many of which make a big difference, and then they notice the impact of their actions, which creates a feedback loop that buoys them to invest further in the change. The sense of possibility initiated by managers provides enough energy for individuals to take small steps towards change; then, witnessing that change increases their sense of possibility, and fosters momentum at a more localised level from there, ultimately contributing to a positive gain cycle.

*“We are noticing that the team are starting to help each other as well, working together as one team across the store.”*

Manager

The positive change trajectory involves multi-level aspects. In addition to the local level, visible changes at the team and store levels also promote the trajectory. For example, team members begin to notice an improvement in communication within their store and a more positive store culture.

*“It’s the little things we now do differently, we’re seeing a change in culture, for everyone not just team leaders. Feedback has been very positive from the team.”*

Manager

*“Our customers are noticing as well; we’re currently tracking at 100% team attitude.”*

In this way, team members experience positive change in their daily work and in all the little things they are doing to make a difference, but they are also aware that the change is happening right across the store. The visibility of the multi-dimensional change in a positive direction propels the trajectory further.

As well as the cognitive aspect of noticing changes, the positive change trajectory encompasses an affective aspect. Team members report positive effects upon noticing that things can and are changing in a way that embraces their contributions. The visibility of change at the local level and the broader level within the store generates feelings of hope, pride, and being valued, further driving the gain spiral.

*“Involvement and listening to team members—they’ve got so many great ideas. Also they feel pretty good when you run with those ideas. Multi-skilling the team, adding skillsets. They also get excited about doing something new, enjoying the new challenge.”*

Manager

From an emotional perspective, when team members witness positive change, they feel more energised and more satisfied with what happens day-to-day. When team members’ little efforts make a big difference at the local level, or they become aware of a more overarching positive change at the store level, it energises them to engage in the next change attempt. Successive change efforts can expand in scope and number as a result of the increasing energetic momentum, meaning that team members also have more to give to the next step.

*“The team have learned so much. Breaking down of all silos—broken down barriers between everyone. It’s fantastic to have the extra tools in our toolbox. It’s the little things.”*

Manager

The cumulative impact of change possibility, change mindset, and positive affect brought about by enacting small positive changes leads team members to think differently, to see new opportunities, and see even greater possibilities for enacting change in ways they would not otherwise have thought.

#### 5.2.5. Change Mechanisms as a Dynamic Process Model

A key feature of the qualitative findings is the interplay between the aggregate dimensions. While having clear and distinct roles to play as individual mechanisms of change, the dimensions are connected to each other in a dynamic change process (as illustrated in Figure 3).

*“We’ve seen a big dynamic change—everyone being open, wants to work together, wants to be multi-skilled; team members here now want a career at [our organisation].”*

Manager

Two dimensions—participatory change and safety net – establish the foundations for successful change. In addition to being enablers at the outset, these two change foundations must be sustained throughout the change process to support the two dimensions of change execution—team unity and positive change trajectory. These change execution dimensions, provided they exist in combination with the change foundations, can turbo-charge the change process.

The interactive and layered process can be seen with the evolution of the safety net. Store members perceive and then engage in the change process within the safety net, which in the early stages is reliant on leaders from within and above the store. Through a combination of the other change foundation and change execution dimensions, such as the increased involvement in participatory change together with the enactment of team unity, the sense of the safety net spreads and becomes less reliant on managers and leaders. What begins as a largely distal mechanism initiated by leaders becomes a dynamic layered process that builds throughout the organisational hierarchy and creates a more localised sense of safety. The change execution dimension positive change trajectory, in turn, reinforces the other aggregate dimensions—it amplifies the effects of the safety signals. Store members perceive the signals and respond by opening up; they start to do new and different things; then in noticing the positive effects their efforts are reinforced, which feels good; and so they become more open to change and continue to invest more in the change because that pattern is actually working.

## 6. Discussion

In this study, we developed an organisational intervention to prevent workplace bullying by addressing the root causes in people management practices [21]; an intervention characterised by deep participation and enabled by co-design as a core process element. In a proof-of-concept study in 10 supermarket stores, using mixed methods and multiple sources of data, we demonstrated the effectiveness of the intervention for preventing bullying and generated knowledge about the mechanisms through which the intervention operates. The quantitative analysis showed that the intervention contributes to a reduction in workplace bullying exposure through a decrease in job demands. The qualitative analysis provided a richer account of how the changes occurred: through two factors that established strong foundations for change—safety net and participation—and two factors that supported successful change execution—team unity and positive change trajectory. Against a backdrop of very few robust research evaluations of organisational interventions for bullying, our findings had significant theoretical and practical implications for advancing workplace bullying prevention.

### 6.1. Theoretical Implications

The first major contribution of our research is demonstrating that participatory organisational interventions can be effectively used as part of a holistic approach to bullying prevention. Specifically, our quantitative data analysis showed that implementing intelligence-led changes to people management practices, when co-designed and co-implemented by employees and managers working together, is associated with reduced bullying risk. The comparative data from stores that did not undertake the intervention strengthens our confidence in this finding. The ten intervention stores recorded reduced internal bullying complaints together with improved customer satisfaction and employee advocacy over the intervention period, while the business-as-usual stores (unaware of the intervention study) recorded deteriorating performance in these three areas over the same period. The intervention stores also had a larger decrease in internal employee relations complaints, and a smaller increase in internal workplace grievances, compared with the business-as-usual stores.

Our study is the first published study, of which we are aware, which evaluates a participatory organisational intervention for bullying that directly addresses bullying antecedents in the work environment (rather than bullying behaviour). Consistent with the work environment hypothesis [22], our findings illustrate that root cause prevention of bullying is feasible and opens the way for a new approach to bullying prevention by improving people management practices. Our findings affirm people management practices as a root cause of workplace bullying, reinforcing the conclusion that the antecedents of bullying are deeper than job design factors and leadership styles, both commonly investigated in the literature. Consistent with the mediation effect through the job demands that we observed, addressing people management practices offers an avenue for making work and organisational changes that presage job design and which can transmit effective leadership into bullying prevention. Overall, our findings encourage efforts to go beyond preventing uncivil behaviour (as per the CREW intervention) [16,17,18] and underscore the potential to move beyond interventions that focus on individual responses to bullying [8]. Though significant effort is involved in designing and conducting intervention research studies of this type, the potential benefits are consequential for advancing sustainable and effective prevention.

Our quantitative analysis suggested that job demands are one mechanism through which our participatory root cause intervention produces reductions in bullying exposure. When people management practices are improved in a collaborative way, employees perceive that their work is less strenuous, demanding, and effortful. In turn, they report less bullying. This finding may reflect a reduction in tension within the organisational system. A decline in job demands lessens the tension between needing to complete work and being able to complete work without too much strain, reduces the tension among employees for resource competition, and alleviates the tension between managers and employees who are recipients of the managers’ decisions in terms of resource allocation and task completion. Such reductions protect employees from energy erosion, interpersonal conflict, and spill-over of aggression. Overall, there is less fertile soil [70] for the onset of bullying.

We discovered that altering job demands was only a small part of the intervention change process. The qualitative findings give a dynamic sense of how changes occur throughout the intervention, highlighting four core ingredients for effective change. First, a safety net must be established, relying initially on safety signals from managers and senior leaders so that employees can become involved in the change process. Within the safety net, participation fosters ownership at multiple levels. Leaders and managers need to drive change but also empower staff to shape the change process in a meaningful way. These two ingredients—safety net and participatory change—represent the foundations for change. When the foundations are established, mechanisms that foster team unity and reinforce a positive change trajectory then enable effective change to be realised. As outlined in the model, these four dimensions interact in a dynamic way. Individually and together, the four aggregate dimensions which we identified in this study represent core process ingredients that emerge through embedding co-design as the fundamental intervention principle. Our detailed account enriches the knowledge based regarding workplace bullying intervention success factors beyond the substance of intervention itself, providing an indication of how to deliver such interventions in practice and offering guidance about the kinds of intervention process effects that can be examined in larger-scale quantitative outcome studies.

### 6.2. Practical Implications

First, our research offers a range of insights into the potential for bullying-specific, prevention-oriented, organisation-focused interventions against workplace bullying, with particular emphasis on improving people management practices through participatory co-design. This approach, which is novel within the field, reduces the burden on individual employees to fight against bullying alone. Instead, it highlights what organisations need to do at the root cause level and what managers and employees can undertake collaboratively. Our findings remind senior leaders of the responsibility for bullying prevention, which lies with organisations, and the possibility of working effectively at an organisational level to complement the proactive role played by employees and managers in creating a healthy and safe work environment. We hope that our evidence concerning the effectiveness of the intervention will motivate organisations to invest in bullying prevention at the root cause level, in addition to bullying response strategies (such as reporting and investigations) and behaviourally oriented prevention strategies (such as policies and training).

Second, our research sheds light on the mechanisms that explain how the intervention works. Such knowledge is critical for developing specific strategies to ensure bullying interventions deliver the desired prevention outcomes. In particular, close attention should be paid to establishing a strong safety net and robust and fair participation structures before moving on to executing the change by cultivating team unity and reinforcing a positive change trajectory. Both these qualitative and quantitative findings indicate that the benefits of participatory organisational interventions can actually move beyond bullying prevention to improve employee advocacy, customer satisfaction, and the overall quality of the work environment. Co-design is a critical success factor underlying these four intervention ingredients. The co-design principle can be realised within different intervention phases from beginning to end, enabling employee participation and active involvement in shaping their work more broadly. Overall, our research offers important knowledge about *how* to effectively design and implement effective bullying prevention.

### 6.3. Strengths, Limitations and Future Directions

In our proof-of-concept study, we collected repeated measurements, performed controlled comparisons between intervention and non-intervention groups, and used multi-source data (self-report and company records). These strengths of our research design increase our confidence in the findings. Even so, several factors limit the conclusions we can draw based on this study. In particular, we evaluated the intervention outcomes in only 10 sites and were unable to match a large number of employees at an individual level; these factors limited our statistical power in the quantitative analysis. In addition, stores were not randomly assigned to the intervention and non-intervention groups. Hence, although we had data from benchmark stores for comparison, it is possible that the intervention stores differed from the non-intervention stores in meaningful ways that ultimately affected our findings (e.g., in terms of the need and readiness for a workplace bullying intervention). In future research, the random assignment of work units would offer a way to mitigate against this possibility, theoretically creating equivalent groups for comparison. Towards this goal, our findings provide a strong signal that it is worthwhile to pursue additional evidence about the effects of organisational interventions for bullying using a robust cluster randomised controlled design.

Though the quantitative analysis did not provide support for job resources as a mediator of the intervention effects, the qualitative analysis generated nuanced insights into the multi-level creation of resources at team and unit levels. Specifically, psychological resources such as ownership fostered by participatory change, relational resources such as team unity, and affective resources such as positive effects generated through a positive change trajectory may all form part of the change process that transmits the effects of the intervention. Future research should further investigate these potential resource-based mechanisms through a quantitative research design, ideally with a multi-level data structure.

Another factor to consider in interpreting our findings is that employees in the intervention stores were aware that the intervention was taking place in their stores. As such, it is possible that managers and employees changed their routine behaviours as a consequence of being observed and assessed during the intervention, not only as a result of the intervention process itself. Therefore, it is possible that the positive changes evidenced in our study may be fostered by participation in an organisational intervention more generally rather than completely reflecting the effectiveness of the specific intervention contents. Given the important role played by intervention participation [50,51] and reinforced by our qualitative analysis, careful consideration should thus be given to how to evaluate intervention effects. Strategies such as using an randomised controlled trial (RCT) design, including a control condition that is aware of their participation plus a group of comparison sites that are unaware of the research, and incorporating objective measures, may all assist in generating insights into this issue. Even within a robust RCT design, it remains important to evaluate intervention process factors together with the assessment of intervention outcomes [23] in order to develop an informative picture of how to prevent bullying as a work environment problem.

## 7. Conclusions

Our study provides evidence of the efficacy of a primary organisational intervention for the prevention of workplace bullying, with a focus on changing the root cause organisational conditions. Our investigation of the mechanisms through which the intervention contributed to a reduction in workplace bullying can inform improvements in the the design and implementation of anti-bullying interventions more broadly, by identifying change foundations and change execution mechanisms. Given the scarcity of sound evidence from bullying intervention studies, our study offers important knowledge on how organisations can effectively prevent and manage bullying at work, redirecting effort and resources from individual employees to the organisation system, and incorporating co-design as a core intervention process principle.

## Figures and Tables

**Table 1:** Evaluation data sources collected during each phase of the intervention.

Intervention Phase	Evaluation Data Sources	
	Quantitative Analysis	Qualitative Analysis (and Waves)
Preparation	--	--
Diagnosis	Risk audit tool assessing people management practices Survey measures of job demands, job resources, and self-reported workplace bullying exposure	--
Solutions	--	--
Implementation	--	Notes from implementation check-in meetings with store (Wave 3)
Evaluation	Risk audit tool assessing people management practices Survey measures of job demands, job resources, and self-reported workplace bullying exposure Internal company complaints data regarding ER cases, grievances, and workplace bullying Internal company data on employee advocacy and customer satisfaction	Focus groups with store leaders and team members (Wave 1) Interviews with HR personnel who supported the intervention (Wave 2)

**Table 2:** Interrater reliability and intraclass correlation coefficient measures.

	Store-Level		Department-Level	
	ICC1	r_wg_	ICC1	r_wg_
Time 1				
People management practices	0.08	0.72	0.10	0.89
Job demands	0.07	0.83	0.09	0.79
Job resources	0.02	0.91	0.01	0.85
Workplace bullying	0.04	0.84	0.03	0.85
Time 2				
People management practices	0.15	0.74	0.09	0.88
Job demands	0.11	0.80	0.12	0.82
Job resources	0.01	0.91	0.09	0.90
Workplace bullying	0.07	0.93	0.11	0.92

**Table 3:** Descriptive statistics and intercorrelations of the study variables.

Variables	M	SD	1	2	3	4	5	6	7	8
Store-level analysis										
Time 1										
1. People management practices	6.63	0.66								
2. Bullying	0.77	0.19	−0.69 *							
3. Job demands	3.19	0.26	−0.67 *	0.73 *						
4. Job resources	3.21	0.17	0.91 **	−0.86 *	−0.78 *					
Time 2										
5. People management practices	7.08	0.70	0.51	−0.56	0.44	−0.76 *	0.63			
6. Bullying	0.52	0.22	−0.37	0.53	−0.41	0.74 *	−0.56	−0.87 *		
7. Job demands	2.95	0.34	−0.06	0.20	0.01	0.63	−0.17	−0.82 *	0.75 *	
8. Job resources	3.39	0.17	0.26	−0.71 *	0.43	−0.52	0.52	0.76 *	−0.77 *	−0.52
Department-level analysis										
Time 1										
1. People management practices	6.57	0.90								
2. Bullying	0.76	0.32	−0.58 **							
3. Job demands	3.23	0.38	−0.44 **	0.65 **						
4. Job resources	3.20	0.29	0.72 **	−0.60 **	−0.48 **					
Time 2										
6. People management practices	7.17	0.91	0.36 *	−0.29	0.18	−0.47 **	0.28			
7. Bullying	0.52	0.36	−0.26	0.17	−0.14	0.31	−0.07	−0.43 *		
8. Job demands	3.01	0.44	0.02	−0.01	−0.09	0.28	−0.09	−0.51 **	0.58 **	
9. Job resources	3.45	0.43	0.15	−0.23	0.13	-0.34 *	0.22	0.46 **	−0.32	−0.14

Notes: *N* = 10 stores at store-level and 34 departments at department-level across Time 1 and 2. * *p* < 0.05. ** *p* < 0.001.

**Table 4:** Mediation results at the department level.

	Job Demands (T2)	Job Resources (T2)	Workplace Bullying (T2)
Controls			
Job demands (T1)	0.07 (0.20)		
Job resources (T1)		0.13 (0.24)	
Bullying (T1)			0.14 (0.15)
Predictors			
People management practices (T2)	−0.23 (0.08) **	0.21 (0.08) **	
Job demands (T2)			0.44 (0.11) **
Job resources (T2)			−0.18 (0.11)

Notes: N = 34 departments. Unstandardised estimates are reported with standard error in the parentheses. ** *p* < 0.001.

## Data Availability

Data are not available due to confidentiality of the organisational records.

## Supplementary Materials

The following supporting information can be downloaded at: https://www.mdpi.com/article/10.3390/ijerph20054373/s1, Table S1: Qualitative data structure (to be included as a Supplementary Material for the Methods section).
