# Supplementary material for: Changing the Underlying Conditions Relevant to Workplace Bullying through Organisational Redesign
Source: Int J Environ Res Public Health. 2023 Feb 28;20(5):4373. doi: 10.3390/ijerph20054373 (PMC10002177; doi:10.3390/ijerph20054373)
Supplement: Supplementary file 1 [file ijerph-20-04373-s001.zip › ijerph-2151659-supplementary.pdf]

## Table S1. Qualitative Paper Data Structure (to be included as a Supplementary Material for the Methods section)

| Data Extracts | 1st Order Concepts | 2nd Order Themes | Aggregate Dimensions (3rd Order) |
|---|---|---|---|
| *"Having [operations] and [HR] partners involved and speaking at the end if they haven't throughout, again just as a signal and a lot of the time they're very supportive."* | Commitment from levels above create more comfort | Management Commitment | Safety Net |
| *"people partner still have a big role, but it's … it's just a people initiative and it gets seen as like a fluffy thing and no operational impact when it's just with the people partners, but as soon as we involve the group managers and the ops managers that's when the stores [store managers] start to realise okay, my actual direct line leaders are involved and this has operational impact. […] But the sooner we bring them on board and we show that to the stores, the more buy in we were getting from the stores."* | People outside the store shape store manager behaviour, focus and engagement | | |
| *"For me it's the collaboration with our team leaders and team members. Amazing how much team leaders want to know about us, not just business related but personal as well." – Team member* | Adopting a whole-person approach to communication | Person-centred approach | |
| *"I definitely think taking the first step in noticing, like you notice that someone's obviously struggling, but taking that first step in actually approaching them…"* | Working to get comfortable with uncomfortable conversations | | |
| *"My job has been to concentrate on the little things like thank yous. The team prefer this personal, natural interaction than to being embarrassed in [meetings]."* | Working with people they way that works best for them | | |
| *"just make sure you're not being defensive, just open yourself up to what is actually happening and what you could do better."* | Team starting to trust | Establishing trust | |
| *"the project itself is a good anchor to keep focussed on team well-being, which is a goal anyway"* | Anchoring focus on wellbeing | | |
| *"and also empowering the team by making just a team member the champion I think would just change the culture on the shop floor level, like on the team member level because they see that one person being able to make changes and they'll know that they can do it too."* | Change Champions drive changes really well | Empowering team members | Participatory Change |
| *"The learning I took out of the project, I know that was the first one, was that you get back what we* | Higher investment in team benefits everyone | | |

| | | | |
|---|---|---|---|
| *invest in the team and we get it back in multiples."* | | | |
| *"And especially checking in with them and listening to them and coming up with an actual plan I think makes them feel heard and valued."* | Getting more input from team members | Listening to team | |
| *"There's a lot more gel, people can come forward and tell us their needs for how to give them the best workplace that we can."* | Opendoor approach | | |
| *"We really explained in the [meeting] that if we don't learn about problems we can't fix them or upskill people."* | Managers view sharing concerns and feedback as helpful | | |
| *"Store manager now starting to help hands on when they are short which is boosting team morale and in picking etc"* | Connection of store manager to team is a key ingredient for making changes | Manager Involvement in Change | |
| *"now we check in with them every day. Our store managers really good with checking in on people whether it's work related or not work related. Yeah, that's pretty much what we've done."* | Top to bottom communication along formal hierarchy | | |
| *"You've got to be the change part at the end of the day, so you can't just wait around for it to happen. If you don't push it, then nobody else is going to. So, it's just important, super, super important."* | Managers proactively leading change is key | | |
| *"we have listened to the team & crossed trained people – its boosted morale, allowed people to meet more team members"* | Improving functional flexibility through cross training | Reducing Silos | Team Unity |
| *"Something to highlight is a lot more unity in store – the team are working now as one team and have each other's backs. This showed on the Friday (lockdown) – everyone jumped in and had each other's backs."* | Sharing responsibilities across departments | | |
| *"[we] ensure good handover so night staff feel part of it too"* | Equal consideration of day and night shift | | |
| *"I mean and it has all worked well this way, but things haven't been easy and nothing's changed as far as budgets and wages, having extra staff on and obviously COVID pressures, none of that's changed but there is just more cooperation"* | Lateral connection & communication | | |
| *"and having people actually jump in and help each other out. I think we were kind of not really a team, but we were like several teams, but now I think we've had that opportunity to really grow and develop, and send people across and cross-train people, so it's now more of that one team mentality as well."* | One Team Mentality | Breaking Down Hierarchy | |
| *"and we cared more about each other because we all realised we were all in the same boat and nobody was completely happy. […]And nobody was going to* | Challenges uniting the team | | |

| | | | |
|---|---|---|---|
| *change it, like nothing was going to change overnight, so we had to change it ourselves."* | | | |
| *"We've taken the divide out of the store – we're not separate departments anymore – it doesn't have to come from me or the ASM either."* | Reduced hierarchy and increased trust | | |
| *"[I] noticed a big change on return from leave"* | Awareness of store culture change | Awareness of Change | Positive Change Trajectory |
| *"There is a noticeable change in mental health awareness, e.g., checking in during lockdown, certain team members who wouldn't check in are now doing it."* | Intervention encouraged noticeable increase in mental health awareness | | |
| *"We have concentrated on our day-to-day conversations, ramping up appreciation during the daily walks."* | Communication climate has changed | | |
| *"We are having regular discussions with TLs and then actioning the issues. It's important to make sure the action happens."* | Visibility of actions reinforces feeling valued | | |
| *"Day to day conversations include "thank you", more one-on-one conversations -[this] effects on culture."* | Little things make a big difference | Change orientated mindset | |
| *"I can see [HR] partners are you know, the way [name] speaks about the project and problems in store, it's a shift of mindset."* | New framing on how to discuss challenges is really helpful | | |
